# Improving Physical Activity and Dietary Behaviours with Single or Multiple Health Behaviour Interventions? A Synthesis of Meta-Analyses and Reviews

**DOI:** 10.3390/ijerph7041720

**Published:** 2010-04-16

**Authors:** Shane N. Sweet, Michelle S. Fortier

**Affiliations:** 1 School of Psychology, University of Ottawa, 125 University Pr., Montpetit Hall, University of Ottawa, Ottawa, Ontario, K1N 6N5, Canada; 2 School of Human Kinetics, University of Ottawa, 125 University Pr., Montpetit Hall, University of Ottawa, Ottawa, Ontario, K1N 6N5, Canada; E-Mail: mfortier@uottawa.ca

**Keywords:** health behaviours, interventions, reviews, meta-analyses, physical activity, dietary behaviours

## Abstract

Since multiple health behaviour interventions have gained popularity, it is important to investigate their effectiveness compared to single health behaviour interventions. This synthesis aims to determine whether single intervention (physical activity or dietary) or multiple interventions (physical activity and dietary) are more effective at increasing these behaviours by synthesizing reviews and meta-analyses. A sub-purpose also explored their impact on weight. Overall, reviews/meta-analyses showed that single health behaviour interventions were more effective at increasing the targeted behaviours, while multiple health behaviour interventions resulted in greater weight loss. This review may assist policies aiming at improving physical activity and nutrition and reversing the obesity epidemic.

## 1. Introduction

Non-communicable diseases (such as diabetes, cardiovascular disease, and cancer) account for 60% of annual deaths worldwide [1]. Specific rates of deaths attributable to chronic disease in Canada and the United States are 89% and 70%, respectively. In addition, rates of obesity have mirrored increases in chronic diseases. In North America, obesity rates have tripled since the 1980s [2]. Recent numbers indicate that the percentage of obese people (Body Mass Index (BMI) > 30) has increased from 13% to 16% between 1994 and 2007 in Canada [3] and 28% to 36% from 1988 to 2006 in the United States [4]. Similar rates have been found in European countries [5,6]. This increase in excess weight has societal impacts since overweight (BMI > 25) and obesity directly influence chronic diseases and account for approximately 58% of diabetes, 21% of ischaemic heart disease and 8–42% of certain cancers [2].

Obesity and chronic diseases have been clearly associated with modifiable health risk behaviours, such as physical inactivity and improper food consumption [2]. However and alarmingly a large percentage of North Americans and Europeans do not engage in the recommended levels of physical activity (30–60 minutes of moderate intensity on most days of the week) [7] and proper dietary behaviours (e.g., eating five to ten fruits and vegetables a day, limiting total fat intake to 20 to 35% of daily intake) [4,8–10]. With regards to North Americans, the percentage of individuals who participate in physical activity 12 times or more per month and eat five fruit and vegetable servings per day has declined from 1988 to 2006 [4], and only 40.6% of the population meet the recommendation for physical activity, 34.4% for percentage of fat intake and 35.1% of fruit and vegetable consumption [11]. When these rates are taken together, only eight to ten percent of the population participate in sufficient amount of physical activity and follow the dietary recommendations [9,11]. Similar results were revealed in Europe. In a national survey of British adults, approximately 76% of the population did not eat enough fruit and vegetables and 66% lacked physical activity [8]. In a survey of Dutch adults, approximately 50% of the sample was not active and 70% ate inadequate amounts of fruit and vegetables [10]. The current trends of sedentary behaviour and unhealthy eating habits have clearly been established in Western society. However, these rates are surprising given that the benefits of participating in the recommended levels of physical activity and adhering to dietary recommendations are well established [12].

Although these benefits have been confirmed for each separate health behaviour [12], research hints that “the combined effects of all diet and physical activity related behaviours that affect health directly and via their effects on obesity have a much larger total effect than any separate pathway” ([13], p. 289). In fact, a recent study revealed that individuals who participated in physical activity and followed a proper diet resulted in a significant reduction of the risk of chronic disease (approximately 65% reduction) compared to those who did not [14]. Therefore, intervening on both physical activity and dietary behaviours at the same time may be a fruitful approach [15], especially since these two health behaviours are highly correlated [16–18] and, as previously mentioned, closely related to overweight, obesity and chronic disease.

Multiple health behaviour interventions have shown to be successful at increasing physical activity and dietary behaviours (such as increasing fibre, fruit and vegetable intake and reducing fat intake) [19,20] as well as decreasing weight [21,22], but there is still some debate regarding their effectiveness.

### Pros and cons of multiple health behaviour interventions

As demonstrated earlier, unhealthy behaviours co-occur and thus targeting more than one behaviour may lead to greater health benefits [23]. In multiple health behaviour interventions, individuals are exposed to a multitude of strategies to change their lifestyle rather than strategies that focus on one health behaviour [15], which is especially important due to the high co-occurrence of health behaviours [24]. In addition, changing one behaviour can facilitate improvements in a second health behaviour, further benefiting the use of multiple health behaviour interventions [2,15,25].

Along with the benefits of intervening on multiple health behaviours, a number of obstacles also exist. The debate is ongoing with regards to whether it is realistic to ask individuals to change more than a single behaviour at a time. Individuals may be confused and feel overwhelmed about changing multiple behaviours [15] and it may push them to quit and return to their unhealthy lifestyle. However, if individuals are capable of changing multiple health behaviours, is there a maximum number of behaviours that they can change simultaneously [15]? Even though there are a number of studies indicating that interventions on single and multiple health behaviours are successful at changing the targeted behaviours, a synthesis of the literature is needed to determine the best approach [15,25].

In fact, a limited number of studies have compared the effectiveness of single (physical activity or dietary behaviours) and multiple health behaviour (physical activity and dietary behaviours) 1 interventions in the same study. Prochaska and Sallis [26] were some of the first researchers to directly compare single *versus* multiple health behaviour interventions. Adolescents were randomized to either a physical activity and nutrition intervention, a physical activity only intervention or a control group. Participants in the intervention groups received a one-time 30-minute tailored intervention and individualized behaviour change or relapse prevention plans. Both intervention groups increased physical activity for boys but not for girls. Although the intervention groups did not find significant results for fruit and vegetable intake, the trends supported both interventions which resulted in increases while the control group decreased their intake. The authors acknowledged that the intervention was only administered once and lacked intensity to foster real change in multiple behaviours. Hence, they called for more intensive interventions to be carried out in order to compare single *versus* multiple health behaviour change.

In line with this call, recent studies have compared these types of interventions for physical activity and dietary behaviours. A study with sedentary women demonstrated that a one-time physical activity only intervention (*i.e.*, print-based motivationally tailored physical activity program) resulted in a greater increase in physical activity than a one-time multiple health intervention (*i.e.*, print-based motivationally tailored physical activity program with nutritional information) [27]. Another intervention study tested whether one-year single behaviour interventions (physical activity only: three time per week supervised endurance-based exercise; diet only group: individually tailored energy restriction plan) or multiple health behaviour interventions (both physical activity and diet) were more successful at decreasing metabolic syndrome in diabetic adults. Although both the physical activity only and the dietary only intervention significantly reduced the prevalence of metabolic syndrome, the multiple health behaviour intervention was more effective at reducing the prevalence than both of the single behaviour interventions alone [28]. With regards to weight as an outcome, another recent study found in a 20-week intervention that a diet only (*i.e.*, individually designed pre-prepared meals) and diet plus exercise group (*i.e.*, treadmill walk 3 times per week) reduced body weight, fat mass and percent body fat in the same magnitude compared to a control group [29]. While interesting, there are too few of these comparative studies to draw any firm conclusions and the results of these studies seem to vary depending on the outcome. Therefore, an in depth analysis of single and multiple health behaviour interventions is needed taking into consideration outcome measured.

### Present synthesis

The purpose of the present paper is therefore to synthesize a set of reviews and meta-analyses that have been conducted on single and multiple health behaviour interventions. Specifically, a separate review of meta-analyses and reviews of physical activity interventions and dietary behaviour interventions will be conducted. Next, a synthesis of meta-analyses and reviews of physical activity and dietary interventions will be carried-out. Lastly, the overall results of the meta-analyses of single behaviour interventions will be compared to the meta-analyses and reviews of multiple health behaviour interventions to determine if one type of intervention is more effective at increasing these behaviours.

Prochaska, Spring and Nigg [23] recently stated that “there exists surprisingly little understanding of some very basic principles concerning multiple health behaviour change” (p. 181). Furthermore, mixed results exist when attempting to determine the effectiveness of multiple behaviour interventions *versus* single behaviour interventions. Therefore, a synthesis of the literature comparing single and multiple health behaviour interventions is warranted to help guide future interventions and increase our understanding of the influence of the different types of behaviour change interventions. In a systematic review of multiple health behaviour interventions, Ebrahim and colleagues determined that these interventions were effective at reducing smoking cessation, but changes in physical activity and dietary behaviour were not reported [30]. Therefore, the current synthesis will focus on reviews and meta-analyses of single and multiple health behaviour interventions which report changes in physical activity and diet. An advantage of synthesising reviews and meta-analyses (*i.e.*, meta-review) is that they provide more evidence than individual studies [31] and thus have been deemed useful [32].

Specifically, the primary objective of this paper is to synthesize a set of reviews and meta-analyses that have been conducted on single (physical activity or diet) and multiple health behaviour interventions (physical activity and diet) and evaluate their effectiveness at changing physical activity and dietary behaviour. Based on the search criteria of the overall objective (*i.e.*, behavioural outcomes), reviews or meta-analyses determining the impact of the interventions on weight will also be retained in order to answer a secondary objective. This secondary objective is to determine whether single or multiple health behaviour interventions are more effective at reducing weight. This secondary objective enabled a comparison of the effectiveness of the interventions across different outcomes. As noted earlier, results from comparative studies directly testing single *versus* multiple health interventions differed based on the outcome. Therefore, answering this will add to the current literature.

## 2. Methodology

### 2.1. Search Strategy

Multiple databases were searched in order to obtain a comprehensive list of reviews and meta-analyses of interventions for physical activity, dietary behaviour and multiple health behaviours. Specifically, electronic searchers were conducted in PsychInfo, Medline, and Cochrane Reviews. Furthermore, reference lists of the reviews/meta-analyses were handsearched to identify potential eligible studies. Other reviews and meta-analyses were added from the author’s personal database.

Search terms to identify the reviews/meta-analyses of interventions for physical activity, dietary behaviour or both were used across the databases. For physical activity, the terms exercise, physical fitness, physical activity, and motor activity were used while the terms diets, nutrition, eating behaviour, energy intake, feeding behaviour and food habits were used for dietary behaviour. To further indentify specific dietary behaviours, keywords such as fruit and vegetables and fat intake were also employed. To target interventions studies, the terms interventions, clinical trials or randomized controlled trial were used. Each search was then limited to reviews and/or meta-analyses in order to isolate those types of articles.

### 2.2. Inclusion Criteria

**Figure 1.**
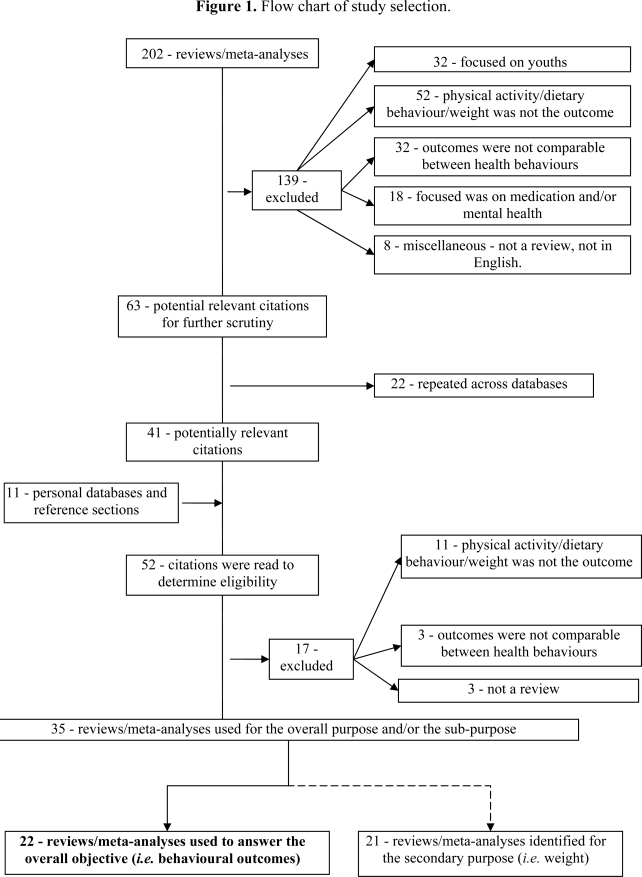
Flow chart of study selection. Note: 8 of the 21 reviews/meta-analyses used to answer the secondary purpose were also reported in the overall purpose.

Reviews and meta-analyses from 2000 and above and those targeting an adult population (18 years or older) were retained. Following Sharma and colleagues’ [33] reasoning, the year 2000 was selected as a cut-off in order to focus on newer reviews and to avoid that the number reviews/meta-analyses be overwhelming. The outcomes of the intervention needed to focus on behavioural outcomes (physical activity and/or dietary behaviours), to answer the overall objective. Since the secondary purpose was to investigate weight as an outcome, a select number of articles were retained if they also focused on weight change. A flow-chart of study selection is presented in Figure 1.

### 2.3. Description of Meta-Analyses/Reviews of Physical Activity Interventions

Across all descriptions, the sum of studies reporting effect sizes and percentages may exceed the total number of reviews/meta-analyses since meta-analyses that provided sufficient information to report a percentage of successful studies were listed twice.

*Behavioural outcomes: Physical Activity.* Sixteen reviews/meta-analyses reported behavioural intervention effects on physical activity levels. Of these 16, five [34–38] provided effect sizes while in 13 [37–49] a percentage of positive effects of the interventions to all reported intervention studies was extracted. The number of interventions studies reviewed in the meta-analyses ranged from 10 to 129, while 4 to 39 interventions studies were included in the reviews.

*Weight Changes.* Four reviews/meta-analyses of physical activity interventions investigated behavioural intervention effects on weight. Of these four, three [36,50,51] reported effect sizes and one [41] was a review. The number of interventions studies included in the meta-analyses ranged from one to 13, while seven intervention studies were highlighted in the review. Across all reviews/meta-analyses, a total of 27 studies were reported.

### 2.4. Description of Meta-Analyses/Reviews of Dietary Interventions

*Behavioural outcomes: Dietary behaviours.* Nine reviews/meta-analyses reported the effects of behavioural interventions on dietary behaviour. Of these nine, three [37,52,53] were reported effect sizes. A percentage of successful studies was reported in eight of the nine reviews/meta-analyses [37,40,43,45,47,53–55]. The number of interventions studies reviewed in the meta-analyses ranged from 6 to 20, while 3 to 17 interventions studies were included in the reviews.

For reviews/meta-analysis on dietary behaviour, it is important to note that six reviews investigated fruit and vegetable intake [40,43,52–55], five looked at fat intake [40,43,52–54], four reported fibre intake [40,52–54] and three reviews explored overall dietary behaviour [37,45,47].

*Weight Changes.* Six reviews/meta-analyses on dietary interventions studied the effects of behavioural interventions on weight. Of these six, one[50] reported an effect size while a percentage of significant and positive effects of the interventions was extracted in five reviews [52,54–57]. Amorim and colleagues [50] only reported the effect size for one study, while 4 to 21 intervention studies were investigated in the other five reviews. Across all dietary behaviours in six reviews/meta-analyses, 58 studies were reported.

### 2.5. Description of Meta-Analyses/Reviews of Multiple Health Behaviour Interventions

*Behavioural outcomes: Physical activity and dietary behaviour.* Six reviews/meta-analyses looked at changes in both diet and physical activity behaviours. Of these studies only one reported an effect size [37] while the percentage of successful studies was found in all six [33,37,43–45,58]. Only one effect size for one study in Eakin *et al.*’s review [37] was reported, while 2 to 6 intervention studies (a total of 27) were highlighted in the other reviews. In addition, three meta-analyses [34–36] compared studies of physical activity only intervention *versus* multiple behaviour interventions. It is important to note that multiple health behaviour interventions in these comparisons were not limited to only physical activity and dietary behaviour interventions.

*Weigh Changes.* Fourteen reviews/meta-analyses examined behavioural intervention effects on weight. Of these 14, eight [50,51,59–64] reported effect sizes while nine [33,44,54,58,60,61,63,65,66] reported the percentage of positive effects of the interventions. Five reviews also tested physical activity and diet *versus* diet alone [50,51,59,61,65]. The number of interventions studies included in the meta-analyses ranged from 1 to 15 for a total of 75, while 74 (range = 3 to 17) intervention studies were highlighted in the reviews.

### 2.6. Data Reporting

*Meta-analyses.* Standardized effect sizes were used to compare the meta-analyses. However, some meta-analyses reported weighted mean difference and thus, based on the data provided by the meta-analyses, a standardized effect size was calculated (Hedge’s g) to allow for comparison.

*Percentage of successful studies.* A percentage was reported based on the findings from reviews and some meta-analyses. The goal was to highlight how many studies found a positive and significant effect of the intervention compared to the total number of studies reviewed. A percentage of 50% indicated mixed results as only half of the studies support the effect of the interventions. Therefore, a percentage of greater than 50% denotes that more than half of the intervention studies demonstrated that the intervention had an effect on the outcome.

## 3. Results—Overall Objective: Behavioural Outcomes

Table 1 provides an overview of each review and meta-analysis used to answer the primary objective at the end of section 3.

### 3.1. Physical Activity Interventions

*Effect size.* Across five meta-analyses, the effect size of physical activity interventions ranged from small to medium (mean = 0.37; range = 0.26 to 0.50) in favour of the intervention group [34–38]. On a dichotomous measure of physical activity, Foster and colleagues [38] revealed an odds ratio of 1.33 favouring the intervention. Two meta-analyses [35,36] investigated the pre-post changes in physical activity in the intervention phase and yielded medium effect sizes (0.41 and 0.57), while finding no effect on the pre-post change of the control group. Even though physical activity interventions reported small to moderate effects, medium size effects were found for the pre-post changes of the intervention groups.

*Percentage of successful studies per review/meta-analysis.* Thirteen reviews of physical activity interventions measuring the percentage of successful studies were reported. Overall, 10 of 13 reviews/meta-analyses [37,39–42,44,46–49] revealed greater than 50% of the intervention studies found a significant and positive effect of the intervention for at least short-term changes. However, only 2 of 4 reviews [37,48] provided support for sustained long-term (greater than six months) changes in physical activity. When looking at individual studies across all reviews/meta-analyses, 66% (139/212; read 139 of 212 hereafter) of all intervention studies demonstrated positive results for increasing physical activity.

### 3.2 Dietary Behaviour Interventions

*Effect size.* Only one meta-analysis examined the effect size for overall dietary behaviour [37], and demonstrated an effect size of 0.74 across four studies. Other studies have reported effect sizes for specific dietary behaviours. Regarding fruit and vegetable intake, a meta-analysis revealed an increase of 1.25 servings/day more for the intervention across 18 studies [52], representing a standardized effect size of 0.80. A second study [53] determined a net change of fruit and vegetables servings per day of 0 to 3.2, favouring the intervention. Based on 10 studies, Pignone and colleagues deemed two studies had a large effect (>1 serving/day), five medium (ranging between 0.3 and 0.9) and three were small to no increase (<0.03 servings/day). The standard effect size calculated for Brunner *et al.* is comparable to the standard which Pignone and colleagues established. Therefore, based on these two meta-analyses, interventions tend to have a medium to large effect on fruit and vegetable intake.

Both of these meta-analyses also looked at the change of fibre intake in grams/day. Both studies differed in their results as Brunner and colleagues [52] showed a weighted mean increase of 5.99 grams/day for the intervention (Hedge’s g = 0.75), while Pignone *et al.* [53] demonstrated a change between 0.3 to 3 grams/day across seven studies representing small to medium effects. Thus, intervention aiming to increase fibre intake are successful, but the degree of their effectiveness is debatable.

Regarding fat intake, Brunner and colleagues [52] also concluded that intervention group had greater decrease in percentage of total fat (4.49%) and saturated fatty acid (2.36%) compared to controls, representing medium to large standardized effect sizes of 0.65 and 0.71, respectively. Pignone and colleagues [53] revealed a 0.9 to 5.3% in saturated fat reduction across 17 studies, with mix results as 6 showed large effects (>3.0%), 5 medium effects (1.3 to 3.0%) and 6 small effects (0.0 to 1.2%).

*Percentage of successful studies per review/meta-analysis.* From eight reviews/meta-analyses, five [37,40,45,53,54] confirmed the effectiveness the interventions when collapsing all dietary behaviours (>50%). From these eight reviews, 67% (101/150) of all reported intervention studies were found to be effective in increasing any type of dietary behaviour. Regarding specific dietary behaviours, four of five reviews/meta-analyses [40,43,53,54] reported greater than 50% of the studies reported increased in fruit and vegetable intake resulting in an average of 67% (24/36) across all interventions. Similar finding were found for fat intake as 3/4 reviews reported support for the effect of the intervention group [40,53,54]. The overall percentage of successful studies for fat intake was 70% (38/54). Increasing intake of fibre was as successful since two of three reviews reported greater than a 50% success rate [40,53] yielding an overall percentage of 56% (9/16).

### 3.3. Multiple Health Behaviours Intervention for Physical Activity and Diet

*Effect size.* No meta-analyses investigated the influence of multiple health behaviour interventions on physical activity and dietary behaviour. However, Eakin and colleagues [37] reported an effect size of 0.86 for one study. Even though no direct meta-analysis was conducted, three meta-analyses [34–36] compared the effect sizes of physical activity only and multiple health behaviour interventions. All three meta-analyses found that physical activity only interventions (ES = 0.39; 0.57; 0.47, respectively) had larger effect sizes than multiple health behaviours (ES = 0.23; 0.38; 0.32, respectively) at increasing one’s physical activity level.

*Percentage of successful studies per review/meta-analysis.* Six reviews reported on the effect of multiple health interventions at increasing physical activity and dietary behaviour and only one found greater than 50% support [37]. On average only 41% (11/27) of the interventions that targeted physical activity and dietary behaviours where effective at changing both behaviours. However, three multiple health behaviour interventions also took into account changes in at least one behaviour and two of the three review/meta-analysis revealed greater than 50% success rate [33,45] and 64% (7/11) success rate across all individual studies.

## 3.4. Comparing Single and Multiple Health Behaviour Interventions for Physical Activity and Diet

Slightly larger effect sizes and a higher percentage of studies supporting the intervention lend weight to single health behaviour interventions over multiple health behaviour interventions. Specifically, physical activity interventions revealed small to moderate effect size and 66% overall support for the intervention, dietary behaviour showed medium to large effect sizes and 67% support. These effects were greater than those found for multiple health behaviour interventions that reported small effect sizes and only 41% of the studies supporting the intervention. Furthermore, multiple health behaviour interventions were more effective at changing one behaviour rather than both behaviour simultaneously. Therefore, focusing on changing a single behaviour is a more fruitful approach than attempting to change multiple behaviours simultaneously.

## 4. Results—Sub-Purpose: Weight changes

Table 2 provides an overview of the reviews and meta-analyses used to answer the secondary purpose at the end of section 4.

### 4.1. Physical Activity Interventions for Changes in Weight

*Effect size.* Only one of three meta-analyses highlighted a significant difference in weight loss between the intervention group and control group. Specifically, Shaw *et al.* [51] demonstrated that individuals in the intervention group lost 2.03 kg more than the control group (Hedges’ g = 0.58), while Conn and colleagues [36] revealed an effect size of 0.07 and Amorim *et al.* [50]showed no effect between the control and experimental group.

*Percentage of successful studies per review/meta-analysis.* Only one review [41] presented sufficient information to calculate the percentage of positive effects of the interventions. With regards to weight, all four studies in the review revealed a difference favouring the intervention group. However, only three of seven studies demonstrated a difference in BMI.

### 4.2. Dietary Behaviour Interventions for Changes in Weight

*Effect size.* Only one meta-analysis [50] reported effect size for dietary intervention and revealed a weighted mean difference of 1.7 kg favouring the intervention, which translated to a very large effect (Hedge’s g of 5.76).

*Percentage of successful studies per review/meta-analysis.* Five reviews presented the percentage of successful studies. Only 1/5 review revealed that more than 50% of the interventions were successful [56]. Overall, most studies did not support the use of dietary intervention for weight loss as only 40% (23/57) of the interventions reviewed reported positive effects of the interventions. However, Rolls and colleagues [55] revealed that in four interventions that mixed a weight loss component with the dietary intervention resulted in greater weight loss.

### 4.3. Multiple Health Behaviour Interventions for Changes in Weight

*Effect size.* Eight meta-analyses reviewed the effect of diet plus physical activity intervention on weight loss and/or BMI [50,51,59–64]. Some meta-analyses compared diet plus physical activity intervention group to a control group or to a diet only group. With regards to diet and physical activity *versus* control group, four meta-analyses [50,62–64] reported a greater decrease in weight for the intervention group (*M* = −3.80 kg; range = −1.91kg to −6.74kg), resulting in an average standardized effect size of 0.81 (range = 0.01 to 1.79). Norris *et al.* [62] reported weighted mean differences which included single and multiple health behaviour interventions. Therefore, a standardized mean difference effect size was calculated only including the multiple health behaviour interventions.

When comparing diet and physical activity against diet only groups, four meta-analyses [50,51,59,61] showed an average weight loss of 1.5kg (range = 0.3 to −6.7kg), translating to a standardized weighted mean difference of 0.18 (range = −0.49 to 0.51) favouring the intervention. However, one meta-analysis [50] found an effect size of 0.49 favouring the control group, but this effect size was only based on one study. When removing this result, a larger standardized mean difference of 0.40 was revealed.

Four meta-analyses [51,60,63,64] also reported weighted mean differences for BMI. Three meta-analyses demonstrated a lower BMI score for those in the intervention group compared to a control group (M = −0.82; range = −0.31 to −1.5) translating to a standardized mean difference of 0.52 (range = 0.31 to 0.97). Another meta-analysis [51] revealed an effect size of 0.21 in favour of the intervention group over a diet only group.

*Percentage of successful studies per review/meta-analysis.* Six of nine reviews had a percentage of over 50% [33,58,61,63,65,66]. A reduction in weight (BMI and weight) for the intervention group was found in 69% (53/77) of the studies. However, when separating BMI and weight, interventions focusing on diet and physical activity had greater success at changing weight (72% of studies) than BMI (54% of studies). Two reviews [61,65] also tested physical activity and diet intervention *versus* diet only on weight changes. Both reviews found perfect support as 100% (7/7) of the interventions on physical activity and diet showed greater weight loss than diet alone.

### 4.4. Comparing Single and Multiple Health Behaviour Interventions for Changes in Weight

Compared to single health behaviour interventions, multiple health behaviour interventions were more effective at reducing weight. Although one review showed support for physical activity intervention, only one of three meta-analyses revealed an effect size supporting the intervention. In the same vein, reviews and meta-analyses did not provide support for dietary behaviour interventions although one meta-analysis provided a large effect. In contrast, 69% of the studies found in the reviews/meta-analyses favoured the combined physical activity and diet interventions. A large effect size was also revealed. In addition, the majority of reviews/meta-analyses for multiple health behaviour interventions demonstrated that the physical activity and diet interventions are more effective at reducing weight compared to diet alone, while moderate effect size was also found for this comparison. However, weaker results were found for changes in BMI.

## 5. Discussion

The primary objective of this review was to investigate, through a synthesis of reviews and meta-analyses, whether single or multiple health behaviour interventions were more effective at changing physical activity and dietary behaviour. To our knowledge, this is the first meta-review to attempt to determine differences between these interventions. A secondary objective was to compare the effectiveness of single and multiple health behaviour interventions on weight loss.

Results for the primary objective revealed that single behaviour interventions were more effective than multiple health behaviour interventions in promoting physical activity and changing dietary behaviour. First, the majority of physical activity only interventions were effective at increasing physical activity and resulted in a moderate size effect. In a review of reviews, Eakin *et al.* [39] also concluded strong support for physical activity interventions. Second, dietary behaviour only interventions revealed similar results as most studies reported moderate effect sizes. Specifically, the largest effects and highest percentage of support were found for interventions targeting fruit and vegetable intake. Although no meta-analyses on multiple health behaviour interventions directly investigated changes in physical activity and dietary behaviour, a select few compared single behaviour interventions to multiple health behaviour which revealed small effects. The reviews on multiple health behaviour interventions demonstrated mixed results regarding their effectiveness at changing physical activity and dietary behaviour. The results from this synthesis support findings from a recent intervention study that tested single *versus* multiple health behaviour intervention in the same study [27]. Dutton and colleagues found that the physical activity only intervention significantly increased the participants’ physical activity levels compared to a physical activity and diet group as well as a control group. The authors also investigated changes in dietary behaviours across the three groups and found that dietary behaviours did not significantly differ between groups [27]. Therefore, the physical activity and diet group did not significantly differ in physical activity and dietary behaviour compared to the other groups, thus providing support for the results of this synthesis on multiple health behaviour interventions. Accordingly, one can conclude that single health behaviour interventions are more effective at changing specific health behaviours than multiple health behaviour interventions.

In addition, the results of this review lends further support to Nigg and colleagues’ [15] hypothesis that changing multiple health behaviours simultaneously may be burdensome for some individuals, thus decreasing the effectiveness multiple health behaviour interventions. These interventions may also lead to the dilution of the intervention whereby single behaviour intervention may delve deeper into the issue at hand and multiple health behaviour interventions might attempt to “accomplish too much” [24]. In Prochaska and Sallis’ study [26], individuals in the multiple health behaviour intervention rated this intervention as being too long, supporting that participants may feel overwhelmed with all the information presented to them. Based on these concerns and the results of the current review, targeting one health behaviour at a time may be more effective than focusing on multiple health behaviours if one aims to change a specific health behaviour.

Regarding the weigh loss objective, multiple health behaviour interventions were more effective at reducing weight compared to single behaviour interventions. First, reviews and meta-analyses from physical activity only interventions revealed very weak support for their effectiveness on weight loss. This finding is congruent with the current literature on physical activity and weight loss [67,68] as physical activity is hypothesized to play a more important role in weight maintenance rather than weight loss [69,70]. Second and contrary to expectations, dietary behaviour interventions did not result in significant weight loss either, but this result could be due to the fact that the focus of this synthesis was not on weight loss. Regardless, the result was surprising as changes to dietary behaviour have been linked to weight loss [70]. Third, the majority of the reviews and meta-analyses in this review supported the effectiveness of multiple health behaviour interventions at reducing weight. Large effect sizes further evidenced multiple health behaviour interventions’ effectiveness for weight loss. A sub-set of reviews also compared diet alone *versus* diet and physical activity intervention arms and showed that the diet and physical activity arm had a slightly larger impact than diet alone. Recent reviews support this statement and conclude that physical activity and dietary interventions should continue to be the focus for weight loss as they are more effective than either behaviour alone [68,71]. Furthermore, including physical activity with a dietary behaviour intervention for a weight loss purpose produces additional health benefits such as reduction in abdominal adiposity [29] and cardiometabolic risk factors that would not be seen with diet only interventions [71].

Results for both purposes and from comparative studies seem to indicate that the effects of single and multiple health behaviours interventions differ as single health behaviour influence the specific behaviours while multiple health behaviours has a greater impact on weight. One hypothesized explanation is that multiple health behaviour interventions could result in small, non-significant improvements in both health behaviours, leading to the conclusion that multiple health behaviour interventions are not effective at changing behaviour. However, these small improvements in both health behaviours may produce significant weight loss. This hypothesized explanation should be explored in future studies, as this result remains perplexing. Furthermore, these reviews/meta-analyses focused on reporting change for each individual behaviour. Prochaska, Velicer, Nigg and Prochaska [72] highlight the limitation to using this approach in multiple behaviour interventions (*i.e.*, increased type one error; difficult to compare across conditions; no overall effect of intervention). Consequently, they proposed new methods to investigate change across multiple behaviours to allow for easier interpretability and comparison between single and multiple behaviour interventions. These new methods (*i.e.*, combining change scores, creating an index score, expanding the impact formula, and using overarching measure of change) need to be tested and compared in multi-behavioural interventions to truly assess the behaviour change effects. It is therefore urged that new multi-behavioural interventions test these new methods of assessing multiple behaviour change.

To our knowledge, this review is the first that attempted to determine the effectiveness of single *versus* multiple health interventions on physical activity and dietary behaviour using a synthesis of reviews and meta-analyses (*i.e.*, a meta-review). The use of a meta-review also allowed for a larger scale synthesis than a review or meta-analysis. Despite the strengths and novelty of this review, limitations still exist. First, these results should be interpreted with caution as more reviews and meta-analyses are needed for multiple health behaviour intervention targeting physical activity and dietary behaviour, in order to confirm the findings of this synthesis. Second, specific physical activity (*i.e.*, walking, running, resistance training) and dietary (*i.e.*, fat intake, fruit & vegetable intake) behaviours were not teased out in this synthesis, therefore a review or meta-analysis investigating the impact of interventions on specific behaviours could reveal interesting results. Third, the focus of this review was not on weight loss. The weight loss results were based on sub-analysis using reviews and meta-analyses found from the search criteria of the overall objective which centered on behavioural outcomes. Therefore, a meta-review focusing on weight loss could find different results. Fourth, a publication bias may exist in this meta-review as only published reviews/meta-analyses were presented and some of the standards for study inclusion in a review are strict, thus limiting the numbers of studies reported. Since reviews and meta-analyses can reduce the specificity of individual studies, some have argued that a review of meta-analyses and reviews further limits the specificity of the individual studies. However, other meta-analyses have summarized findings from meta-analyses in particular areas of research such as social psychology [32], and health related fields [31,73] thus supporting this type of review.

### Next steps in behavioural health interventions?

Interventions testing single *versus* multiple health behaviour interventions are still far and few between. Therefore, more studies directly comparing these interventions in the same study are needed to help determine which are more effective in order to confirm the results of this review. A recent line of research began testing simultaneous and sequential multiple health behaviour interventions to determine if spacing the behavioural interventions for physical activity and diet would result in greater adoption than changing health behaviours at the same time. Summarizing this novel but limited literature revealed mixed results. Specifically, two studies noted no difference between both types of interventions [74,75], one favoured the simultaneous group [76], while two other studies supported sequential interventions [77,78]. Therefore more research is needed in this new area to understand the proper sequencing or timing of the health behaviour interventions.

On a theoretical note, experts in the field have begun to recommend that theoretical frameworks serve as a guide in the development of interventions [79,80]. However, many interventions are not following this recommendation as less than 30% of interventions have properly applied theory to guide their intervention and tested the impact of the intervention on the respected theoretical constructs [81]. Theoretical variables have been shown to be important in multiple health behaviour research especially regarding motivational transference. Increased confidence in changing one behaviour has been shown [82] to lead to greater success in changing a second behaviour. Therefore, a closer examination of mediating variables should be conducted to identify which variables are important for each specific behaviour and if increases in a particular theoretical construct facilitates adoption of a second health behaviour.

Based on this review and other recommendations we suggest more empirical studies comparing single *versus* multiple health behaviour interventions and more reviews and meta-analyses on multiple health behaviours. Specific to empirical studies, we recommend that they use randomized control design, assess the behaviours over time, use objective measures, have a theoretical foundation and test psychological mediation effects. A meta-analysis determining the theoretical constructs associated with changes in behaviour and weight would be a strong contribution, especially since some evidence exists on the influence of mediating variables [23]. A different line of research could observe if the effectiveness of health behavioural interventions differ across various populations such as low social economic status individuals and gender. A meta-analysis could help determine the impact of the interventions across different populations. In addition, future reviews could investigate the influence of single and multiple health behaviours on other health outcomes such as indicators of diabetes, cancer, or cardiovascular disease.

## 6. Conclusion

Based on the results of this review, interventions aiming to change a specific behaviour should focus their intervening on a single behaviour rather than potentially overloading their patients with multiple behaviour changes. In contrast, if one aims to change weight, findings from this review recommend that both physical activity and dietary behaviour should be targeted. Therefore, it is important for researchers to gear their interventions based on the desired outcome.

As described in the introduction, physical inactivity, improper diet and excess weight are key modifiable risk factors associated with a large number of chronic diseases. Regardless of the type of intervention, one of the three listed risk factors was improved. However, from a policy standpoint, one could conclude that multiple health behaviour interventions are more cost effective due to their influence on weight. As a result, having a clear understanding of the effectiveness of these types of interventions and their influence on various outcomes is crucial in order to help guide and orient health care policies.

## Figures and Tables

**Table 1.** Summary of reviews/meta-analyses for behavioural outcomes.

Review/Meta-Analysis	Year	Number of Articles	Percentage	Effect size

*Physical Activity*				

Conn *et al.* [34]	2002	43	N/A	0.26
Conn *et al*. [35]	2008	62	N/A	0.35
Conn *et al.* [36]	2009	129	N/A	0.45
Eakin *et al.* [39]	2005	9 (all reviews)	67%	
		All 9 reported on short-term PA	67%	N/A
		All 9 reported on long-term PA	0%	
Eakin *et al.* [37]	2007	16 (all studies)	69%	0.5 (8 of 16 studies)
		15 of 16 reported on short-term PA	47%	
		6 of 6 reported on long-term PA	100%	
Foster *et al.* [38]	2005	29 (all studies)	31%	
		19 (continuous PA)	37%	0.28
		10 (categorical PA)	20%	1.33 (Odds Ratio)
Janer *et al.* [40]	2002	15	67%	N/A
Kavookjian *et al.* [41]	2007	18	78%	N/A
Khan *et al.* [42]	2002	39	74%	N/A
Kroeze *et al.* [43]	2006	4	25%	N/A
Neville *et al.* [44]	2009	8	63%	N/A
Norris *et al.* [45]	2001	6	50%	N/A
Ogilvie *et al.* [46]	2007	18	89%	N/A
Shilts *et al.* [47]	2004	8	75%	N/A
Tulloch *et al.* [48]	2006	20 (all studies)	75%	
		9 of 20 reported on short-term PA	78%	N/A
		13 of 20 reported on long-term PA	69%	
van der Bij *et al.* [49]	2002	22 (all studies)	64%	
		9 of 22 reported on long-term	33%	N/A

*Dietary Behaviours*				

Brunner *et al.* [52]	2007	F&V : 18		F&V : 0.80
		Fiber : 9		Fiber : 0.75
		FI : 20	N/A	FI : 0.65
		FI : 12 (saturated)		FI : 0.71 (saturated)
Eakin *et al.* [37]	2007	6	83%	0.74 (4 of 6 studies)
Janer *et al.* [40]	2002		All behaviours: 66%	
		V: 7	V: 86%	
		F: 7	F: 57%	N/A
		FI: 10	FI: 60%	
		Fiber: 5	Fiber: 60%	
Kroeze *et al.* [43]	2006		All behaviours: 38%	
		FI: 5	FI: 20%	N/A
		F&V: 3	F&V: 67%	
Norris *et al.* [45]	2001	11	82%	N/A
Pignone *et al.* [53]	2003		All behaviours: 91%	
		FI : 17	FI (only 15 studies reported significance): 93%	FI : N/A, but 6 were rated as having a large effect, 5 medium, 6 small
		F&V : 10	F&V (only 4 of 10 studies reported significance): 75%	F&V: N/A, but 2 were rated as having a large large, 5 medium, 3 small
		Fiber : 7	Fiber (only 3 studies reported significance): 100%	Fiber: N/A, but 5 were rated as having a medium and 2 a small effect.

Povey *et al.* [54]	2007		All behaviours: 62%	
		FI: 14	FI: 71%	N/A
		FI: 10 (saturated)	FI: 70% (saturated)	
		F&V: 3	F&V: 100%	
		Fiber: 8	Fiber: 38%	
Rolls *et al.* [55]	2004	F&V: 12	F&V: 50%	N/A
Shilts *et al.* [47]	2004	4	50%	N/A

*Physical Activity and Diet*				

Blue & Black [58]	2005	6	50%^a^	N/A
Eakin *et al.* [37]	2007	4	75%^a^	.86 (1 study)
Kroeze *et al.* [43]	2006	6	17%^a^	N/A
			33%^b^	
Neville *et al.* [44]	2009	6	33%	N/A
Norris *et al.* [45]	2001	3	33%^a^	N/A
			100%^b^	
Sharma [33]	2007	2	50%^a^	N/A
			100%^b^	

Notes: N/A = Not applicable as either an effect size was not calculated or a percentage could not be extracted. PA = physical activity. FI = fat intake. F&V = fruit and vegetable intake. Short-term = < 6 months. Long-term = > 6 months.

a Changes in both physical activity and dietary behaviour.

b Changes in either physical activity or dietary behaviour.

**Table 2.** Summary of reviews and meta-analyses for weight changes.

Review/Meta-Analysis	Year	Number of Articles	Percentage	Effect Size

*Physical Activity*				

Amorim *et al.* [50]	2007	1	N/A	0.00
Conn *et al.*^a^ [36]	2009	13	N/A	0.07
Kavookjian *et al.*^a^ [41]	2007	4	100%	N/A
		7 (BMI)	43% (BMI)	
Shaw *et al.* [51]	2006	2	N/A	0.58

*Dietary Behaviours*				

Amorim *et al.* [50]	2007	1	N/A	5.76
Brunner *et al.*^a^ [52]	2007	21	38%	N/A
Hooper *et al.* [56]	2004	5	60%	N/A
Povey *et al.*^a^ [54]	2007	6	17%	N/A
		5 (BMI)	40%	
Rolls *et al.*^a^ [55]	2004	16	44%	N/A
Rooney *et al.* [57]	2007	4	50%	N/A

*Physical Activity and Diet*				

Amorim *et al.* [50]	2007	4	N/A	1.79^i^
		1		−0.49^ii^
Blue & Black^a^ [58]	2005	11	73%^i^	N/A
Chaston *et al.* [65]	2007	3	100%^ii^	N/A
Curioni *et al.* [59]	2005	6	N/A	0.2^ii^
Hawthorne *et al.* [60]	2008	3 (BMI)	0% i (BMI)	0.31 i (BMI)
Ketola *et al.* [66]	2000	17	71% i	N/A
Neville *et al.*^a^ [44]	2009	6	50% i	N/A
Nield *et al.* [61]	2007	4	100%^ii^	0.51^ii^
Norris *et al.* [63]	2005	9	83% i	0.77^i^ (7 studies)
		6 (BMI)	67% i (BMI)	0.97^i^ (BMI)
Norris *et al.* [62]	2005b	8	N/A	0.01^i^
Orozco *et al.* [64]	2008	7	N/A	0.66^i^
		6 (BMI)		0.57^i^ (BMI)
Povey *et al.*^a^ [54]	2007	6	33%^i^	N/A
Sharma^a^ [33]	2007	8	88%^i^	N/A
		4 (BMI)	75%^i^ (BMI)	
Shaw *et al.* [51]	2006	15	N/A	0.48^ii^
		6 (BMI)		0.21^ii^ (BMI)

Note: N/A = Not applicable as either an effect size was not calculated or a percentage could not be extracted. PA = physical activity. BMI = Body Mass Index.

a Review/Meta-analysis was used in overall objective (*i.e.*, behavioral outcome).

i Diet and physical activity group *versus* control group.

ii Diet and physical activity group *versus* diet only group.

## Footnotes

1 Unless otherwise indicated, reference to single health behaviour will correspond to either physical activity or dietary behaviours while physical activity and dietary behaviours will represent multiple health behaviours. Although we recognize that physical activity and dietary behaviours can be comprised of multiple sub-behaviours (*i.e.*, walking for physical activity, fat intake for dietary), the labels of physical activity and dietary behaviours will reflect the behaviours as a whole unless specified differently.
